# Evaluation of Long-Term Performance of a Solar Home System (SHS) Monitoring System on Harsh Environments

**DOI:** 10.3390/s19245462

**Published:** 2019-12-11

**Authors:** Ascensión López-Vargas, Manuel Fuentes, Marta Vivar

**Affiliations:** 1 Water and Energy Group, IMDEA Water Institute, 28805 Alcalá de Henares, Spain; 2 Grupo IDEA, EPS Linares, University of Jaén, 23700 Linares, Spain; mfuentes@ujaen.es (M.F.); marta.vivar@gmail.com (M.V.)

**Keywords:** Internet of Things (IoT), performance, simulation, and modeling, social sensing, optimization, datalogger, monitoring, SHS, harsh environmental conditions

## Abstract

Dataloggers installed in rural regions of developing countries need to be autonomous, robust, and have good recording capacity as they may be exposed to harsh environmental conditions. An extremely hot, dry, and dusty climate can imply additional wear and tear toequipment. A test procedurewas designed and run in a confined space to control climate conditions to test the datalogger. An outdoor campaign lasting more than three years was performed at the Instituto Madrileño de Estudios Avanzados (IMDEA) Water Institute, in Alcalá de Henares (Madrid, Spain) and at the Escuela Politécnica Superior (EPS) Linares (Jaén, Spain) to test the low-cost datalogger under real conditions. The results demonstrated that it was robust and endured extreme weather conditions. In order to avoid the loss of data, a new version with a redundant system based on an SD card was implemented and tested under real conditions.

## 1. Introduction

Grid-connected photovoltaic (PV) systems often involve large budgets, and monitoring devices measure the main parameters to apply the necessary corrective and maintenance operations without strongly influencing the total cost of the installation. However, size and cost aspects are decisive in solar home systems (SHSs). SHSs lack appropriate monitoring and the impossibility of detecting operation and maintenance problems can drastically shorten a system’s lifetime or even the withdrawal of its usage [1].

Millions of SHSs have been installed in remote locations, mainly in isolated regions of Asia, Africa, and Latin America, thanks to electrification programs emerging. Monitoring these PV systems has been identified as one of the key agents that contribute to the success of rural electrification programs [2] as these actions extend the systems’ lifetime and minimize operation failures. In addition, it has also been demonstrated [3] that a lack of information about the performance of SHSs and projects slows down further development and successful dissemination. The cost of commercial dataloggers has decreased in recent years, but it remains too high, and frequently even surpasses the price of the complete stand-alone PV system. As a result, the further development of dataloggers has been required to monitor these PV systems.

For PV application monitoring, open-source platforms, such as Arduino [4,5,6,7,8] or Raspberry PI [9,10,11,12], have been integrated to lower costs, using wireless technologies [13,14,15,16] to transmit data. Dataloggers installed in rural regions of developing countries need to be autonomous and have good recording capacity because SHSs are commonly installed in locations where there is neither an electrical grid nor traditional wired telecommunication networks, and they are often difficult to access by everyday transport. As for modern communications, due to the quality of wired networks in developing countries, Internet services frequently and successively drop. Dataloggers need to be robust as they may be exposed to harsh environmental conditions. Schelling et al. [17] reported that one of the fundamental roadblocks to sustainable and scalable solar electrification is high maintenance costs induced by several factors: High equipment failure rates, poor maintenance/rough usage practices, high fixed maintenance costs due to travel, low user densities in sparsely populated areas, and the severe lack of accountability in the system. For equipment failures, these authors detected that an extremely hot, dry, and dusty climate can affect PV systems with additional wear and tear on equipment. This is an issue of special concern because the climate zones with the highest temperatures (in regions between latitudes N 35°and S 35°) are found in many developing countries in central Africa, the Americas, and South Asia, and extreme climate conditions can affect the correct functioning of the dataloggers installed in rural areas of these countries.

In 2014, Fuentes et al. [18] proposed a low-cost monitoring system designed around open source and free hardware platforms (Arduino^TM^) to monitor PV systems. It was designed especially to be installed in isolated regions or rural areas in developing countries. The datalogger measured up to eight electrical/meteorological parameters and three analog temperatures with a resolution of 18-bits. The datalogger was empirically tested under real environmental conditions and accomplished the accuracy requirements of the IEC standards for PV systems. Later in 2018, an improved prototype of this low-cost monitoring system was developed by López–Vargas et al. [19]. Its main enhancements were: Minimized power consumption, more meteorological parameters measured, improved electrical measurements, and a user-friendly interface was integrated. The new datalogger was designed specifically for SHSs located in remote regions. The prototype was empirically tested by measuring a real, stand-alone PV system with very accurate results; the datalogger proved to be autonomous, have a low-cost, and robust in harsh environments. The data storage system was based on an SD card that required a manual procedure involving a human operator to collect data. This circumstance can be an inconvenience at locations that are difficult to access, and increases maintenance and operating costs.

In 2018, López–Vargas et al. [20] developed a datalogger based on an IoT (Internet of Things) application. These authors stated that integrating IoT allows SHSs to be remotely monitored and the data measured by the datalogger to be visualized using laptops or smartphones. Moreover the use of open IoT cloud platforms minimizes the cost of monitoring systems. 3G Internet connectivity was added. Instantaneous information is received, so the operating and maintenance problems related to the stand-alone PV system can be rapidly detected and solved. Three of these dataloggers were installed at three different locations: Oaxaca (Oaxaca, Mexico), Alcalá de Henares (Madrid, Spain), and the EPS Linares (Jaén, Spain). The authors intensively tested the three dataloggers for a 12-months period to identify the datalogger’s performance when failure occurred. The experimental procedure demonstrated that the installed dataloggers were highly reliable as only four failures were recording over one-year and the origin of most failures was external. They classified the failures found during the experimentation testsas access failures (failed access to the server), power failures, network failures, and equipment failures. The origin of access, power, or network failures is usually external and not linked to the datalogger. Equipment failures comprise the most hazardous failures because they refer to datalogger failures. Such failures include errors such as sensor breakage, errors in the connection system, or even the complete breakdown of the monitoring system. While the other failures (access, power, and network) do not have permanent consequences and only imply operation temporarily stopping, equipment failures can permanently affect some datalogger functionalities and can even render them inoperative. The datalogger located in Oaxaca recorded one error as a result of network outage. The datalogger installed in Alcalá de Henares failed on one occasion due to power shortage. The datalogger in Linares registered two errors: Equipment failure and failed access to the server. The error detected in Alcala did not have any consequences as the datalogger was designed to re-hook and resend data after a power shortage. However, the other recorded failures revealed that some system operation aspects could improve.

**Figure 1:**
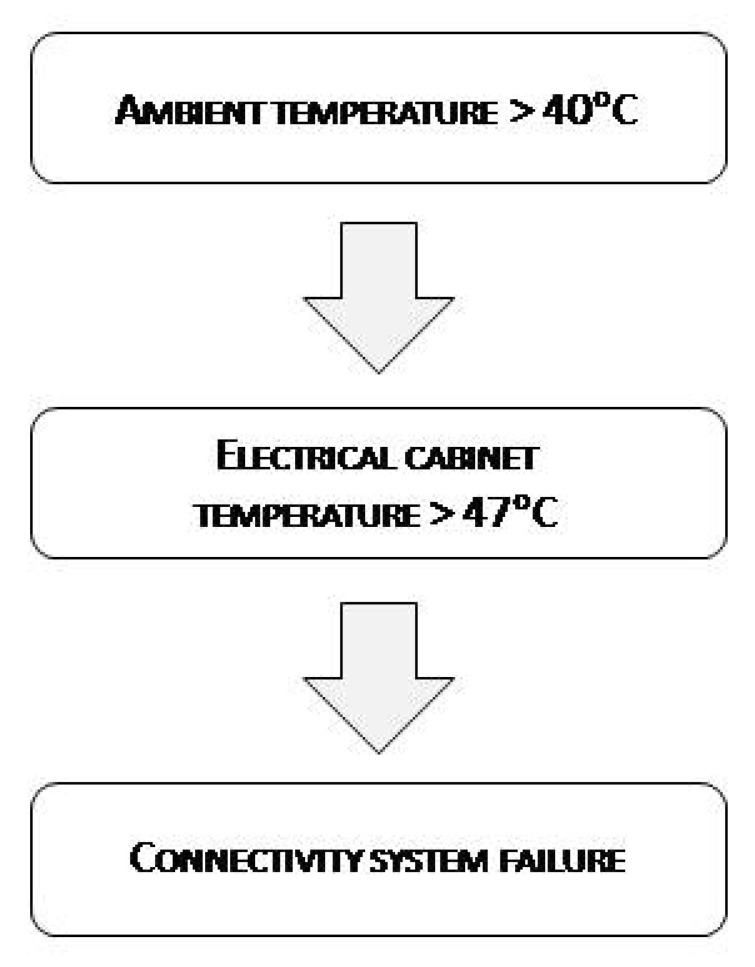
Maximum temperature registered the month before the equipment failure.

The equipment failure was recorded in the datalogger installed in Linares on 29 June 2017 and the system was rendered inoperable and stopped transmitting data. In Linares, PV systems and electronic equipment have to withstand very harsh environmental conditions in summer: Temperatures up to 45 °C, dust storms, and occasional high-speed electrical storms and heavy rain. A refrigeration system formed by two fans was installed, but was not enough. Figure 1 shows the flow diagram with the maximum temperatures registered the month before the failure.

The other major detected problem was the loss of data due to network and access failures. Two monitoring systems were installed in Oaxaca: The Arduino^TM^ datalogger and a “LabJack^TM^ U12 series” commercial datalogger. The commercial datalogger was configured for storing data in a folder shared over the Internet. On 17 June 2017, a failure was detected: Both the Arduino^TM^ monitoring system and the commercial datalogger stopped sending data. On 19 June 2017, both the Arduino^TM^ and the commercial dataloggers started sending data again. The error was classified as a network failure because the commercial datalogger had to be initialized manually to send data if a power failure occurred. Subsequently, networks failures were recorded at the Oaxaca site, and these failures led to a loss of data during fault periods. On 16 November 2017, an access failure to the server installed in Linares was recorded. As maintenance activities were performed, the server installed in Linares was disconnected for short periods of time, affecting all three dataloggers. During these periods, the monitoring systems were unable to connect to the server and data were lost.

The ultimate goal of this work is to optimize the datalogger, by running the system at stream temperatures and reinforcing the data storage system. The thermal evaluation of the system to guarantee the reliability of the long-term datalogger was made by running an indoor test(temperature cycling tests) and evaluating the dataloggers under real conditions for three-years. Further, the main optimization strategies included designing and testing a new backup storage system.

The structure of this paper is as follows. Section 2 presents the indoor temperature stress tests and discusses the results, while Section 3 presents the result of the long-term performance of the system working under harsh environmental conditions. Section 4 presents optimizations. Section 5 presents a discussion of the results. A summary of the conclusions is included in Section 6.

## 2. Indoor Temperature Stress Tests

**Figure 2:**
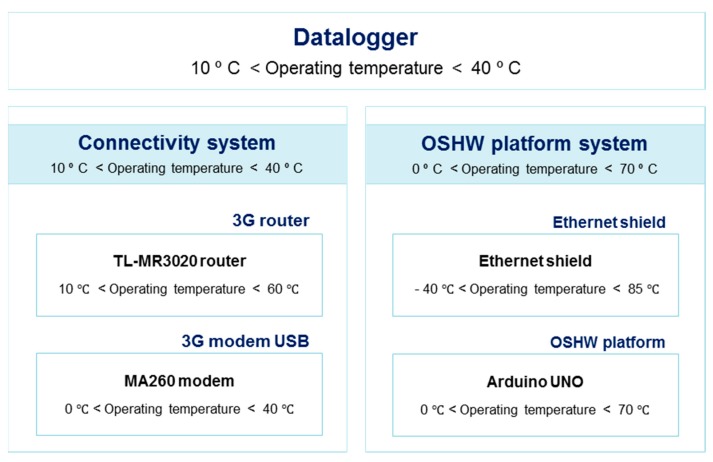
Global diagram of the monitoring system: Operating temperatures.

Figure 2 shows the configuration of the entire monitoring system by focusing on operating temperatures. Of the numerous options to connect the Open Source Hardware (OSHW) platform to the Internet using 3G, the TL-MR3020 router from TP-LINK was selected given its potential to configure Wi-Fi and 3G transmission systems at a low price. This device requires an external 5VDC/1A power supply and the temperature range operates from 10 °C to 60 °C with relative humidity conditions within a range from 10% relative humidity (RH) to 90% RH (non-condensing). As the router has an on-board interface USB 2.0 port, the 3G operating mode was configured by providing an Internet connection via a 3G USB stick modem. For this purpose, the MA260 modem from TP-LINK was selected. The USB modem operating temperature range was 0–40 °C. Thus, the operating temperature of the entire system (router and modem) was limited by the most restricted values: 10–40 °C. The operating temperature of the connectivity system was the most limited.

As the system’s maximum operative conditions were reached, by all accounts it would seem that an equipment failure in the connectivity system was due to high temperatures. After inspecting the system, it was found that, in fact, the data delivery system (consisting of the nanorouter and modem) stopped working. The connectivity system was not broken, but it was necessary to reset the nanorouter and reconfigure it manually.

### 2.1. Experimental Evaluation of High Temperature Testing

A test procedure was designed to check the system’s performance under adverse environmental conditions. The system’s behavior at extreme temperatures was checked to determine if it was the cause of the failure. An experimental procedure that focused on the system’s performance according to temperature and time was run. The objective of the test was to replicate the conditions that could have triggered the failure on 26 June 2017. Performing the text in a confined space allows for the control of times and experiment conditions.

International standard IEC60068 contains a collection of methods for the environmental testing of electronic equipment to assess its ability to perform under environmental conditions, including extreme cold and dry heat. IEC 60068-2-14 [21] provides a test protocol to determine the ability of components, equipment, or other articles to withstand rapid changes in ambient temperature (N test). As this involves the prototype level and the datalogger, which are not yet commercialized, the application of this standard is not required.

To design the experimental procedure, the data measured by the datalogger installed in Linares (Jaén, Spain) were used. The prototype specifically designed for measuring SHSs includes a wide range of climate measures: Irradiance (Gi), ambient temperature (Tamb), humidity (H), wind velocity (W), and rainfall (R). DS18B20 temperature sensors from Maxim Integrated^TM^ (San Jose, CA, USA) were used to acquire the PV module temperature (Tmod), the temperature measured inside the electrical cabinet (Tec) and the PV battery temperature (Tbat). The electrical parameters measured by the low-cost datalogger included a PV generator output voltage (VA), battery voltage (VS), load voltage (VL), PV generator output current (IA), battery current (IS), and load current (IL). The datalogger was installed inside an electrical cabinet.

#### 2.1.1. Test Design

To test the design, an exhaustive study of the data collected in the month prior to the failure recorded in Linares on 28 June 2017 was conducted by paying special attention to the measured temperatures. Table 1 shows the minimum, maximum, and average values of the ambient temperature (Tamb)—the temperature inside the electrical cabinet (Tec) and the humidity (H) recorded in Linares from 1 June 2017 to 28 June 2017.

As shown in Table 1, the electrical cabinet temperature was 6–9 °C higher than the ambient temperature. Thus, the electrical cabinet temperature will be used for the test design.

#### 2.1.2. Selecting Severities

The severity of the test comprises the maximum temperatures, the speed of variation of temperature selected, the time the datalogger was exposed, and the number of cycles that the datalogger was subjected to.

(a) Selecting Temperatures

**Figure 3:**
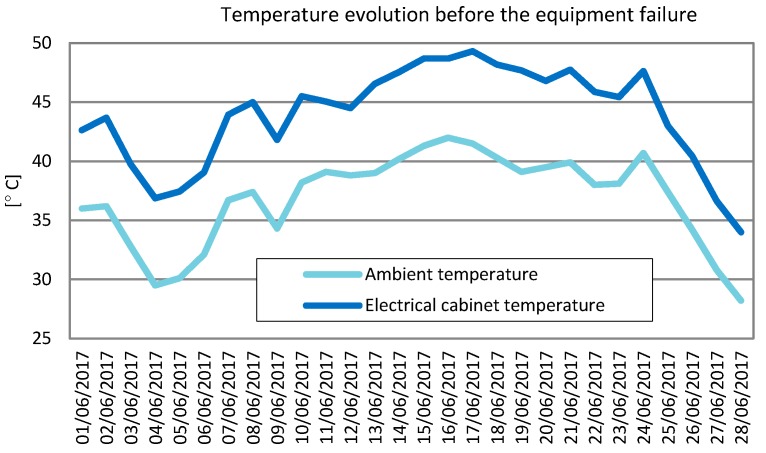
Maximum values of ambient temperature and electrical cabinet temperature measured in June 2017 by the prototype installed in Linares (Jaén, Spain).

Figure 3 shows the monthly variation in the maximum ambient temperature and maximum electrical cabinet temperature. The worst case was recorded on 17 June 2017, when the temperature inside the electrical cabinet reached 49 °C (ambient temperature of 41 °C). For this reason, the temperature that the datalogger was subjected to had to be above 49 °C. The minimum electrical cabinet temperature values fell within the range of 16–27 °C, so this parameter was not decisive in the test.

(b) Speed of Variation Temperature

**Figure 4:**
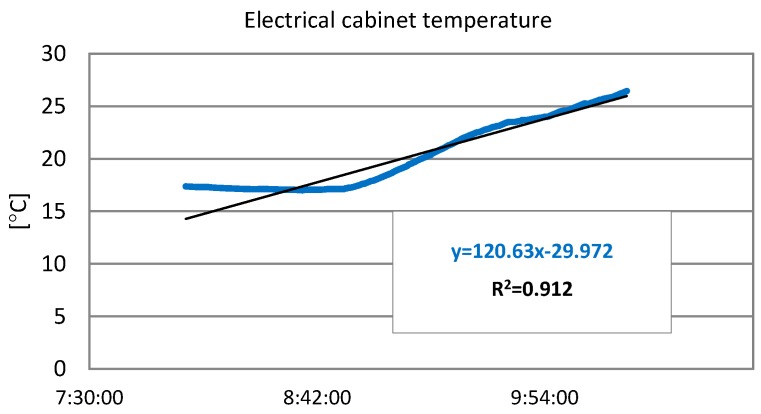
Worst case: Speed of variation temperature measured in Linares on 29 June 2017.

To select the speed of variation temperature, the real data captured by dataloggers before the equipment failure occurred were used. The gradients of curves were studied. Figure 4 shows the worst case, with the most abrupt change in temperature in less time. This was recorded on 29 June 2017. A rise of approximately 0.09 °C/min was recorded. This value corresponds to the maximum speed of recorded variation temperature.

(c) Test Exposure Time

**Figure 5:**
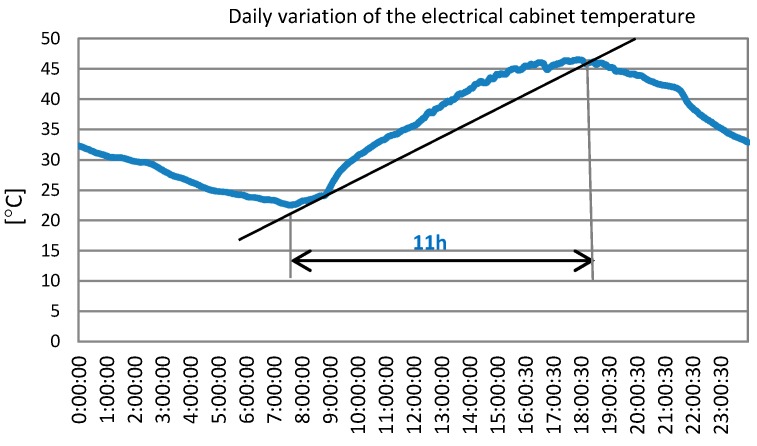
Data recorded in Linares on 13 June 2017.

After studying the cycle of temperatures, the average duration of the cycle of the growing electrical cabinet temperature was 11 h (see Figure 5), which was taken as the exposure time selected for the test.

(d) Number of Cycles

The number of days per year in which the ambient temperature reached 40 °C was limited (the electrical cabinet temperature was 6–9 °C higher than the ambient temperature). In 2017, in Jaén (where the equipment failure occurred, see Table 2), there were 13 days with a maximum temperature reaching 40 °C. Thus, the number of cycles selected for the temperature test was 20 cycles.

### 2.2. Temperature Test in a Confined Space: Setup

A climate chamber (Binder EIW-179) was used for the temperature test. This climate chamber allows for the selection of the set-point temperature as well as the speed of temperature variation until it is reached. The ambient temperature inside the climate chamber was measured by a DHT22 (Aosong Electronics Co., Ltd., Guangzhou, China) digital sensor. The DHT22 sensor measured the temperature and relative humidity [22]. Prior to experimentation, individual test cycles were run to find whether the relative humidity values recorded inside the climate chamber were appropriate, within the range of relative humidity values measured in Linares between 1 June 2017 and 28 June 2017 (Table 1).

**Figure 6:**
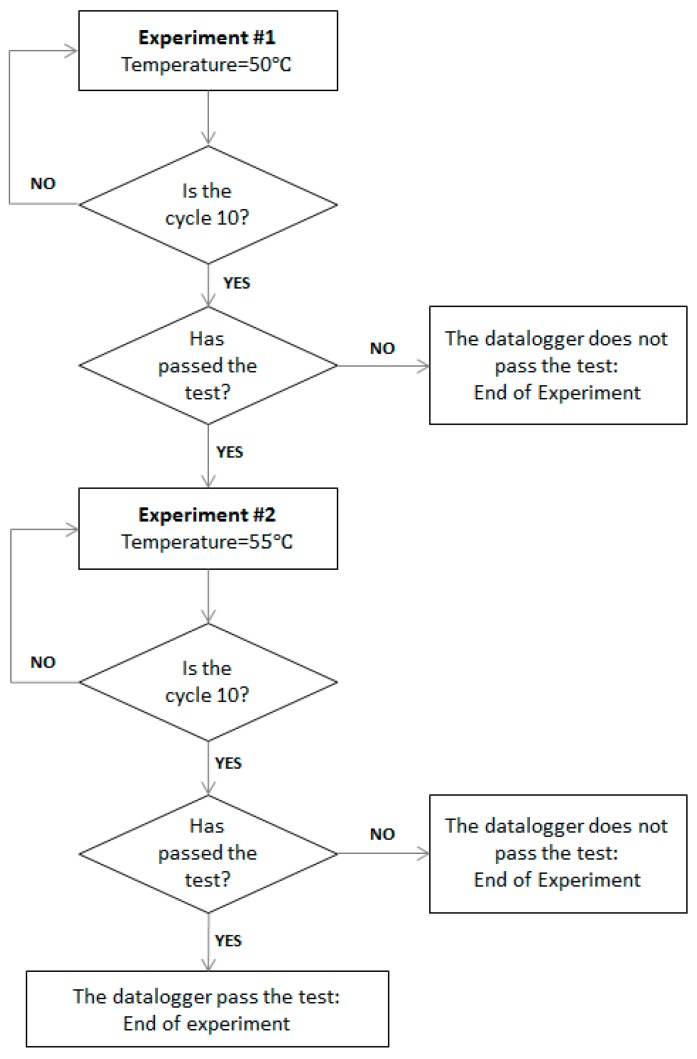
Block diagram of the entire experiment.

Table 3 shows the final values selected to run the temperature test. The maximum recorded rise was approximately 0.09 °C/min. Given the resolution of the employed chamber, the selected speed of temperature variation was 0.1 °C/min. Figure 6 shows the block diagram of the entire experiment, including Experiments #1 and #2. The process was composed of two experiments; each experiment comprised 10 cycles. The maximum temperature used in Experiment #1 was 50 °C; the maximum temperature raised in Experiment #2 was 55 °C. They were consecutive processes: To start Experiment #2, the datalogger had to first pass Experiment #1.

In the indoor experimentation, the environmental parameters (temperature and humidity) were measured. Temperature and humidity [23] were the parameters that had the greatest negative consequence in electronic equipment. Due to the dimensions of the climatic chamber, it was not possible to include a PV module to measure electrical parameters. This aspect did not act to the detriment of the tests because the study was mainly focused on connectivity, and the measurement of climatic parameters allowed for the evaluation of the datalogger.

**Figure 7:**
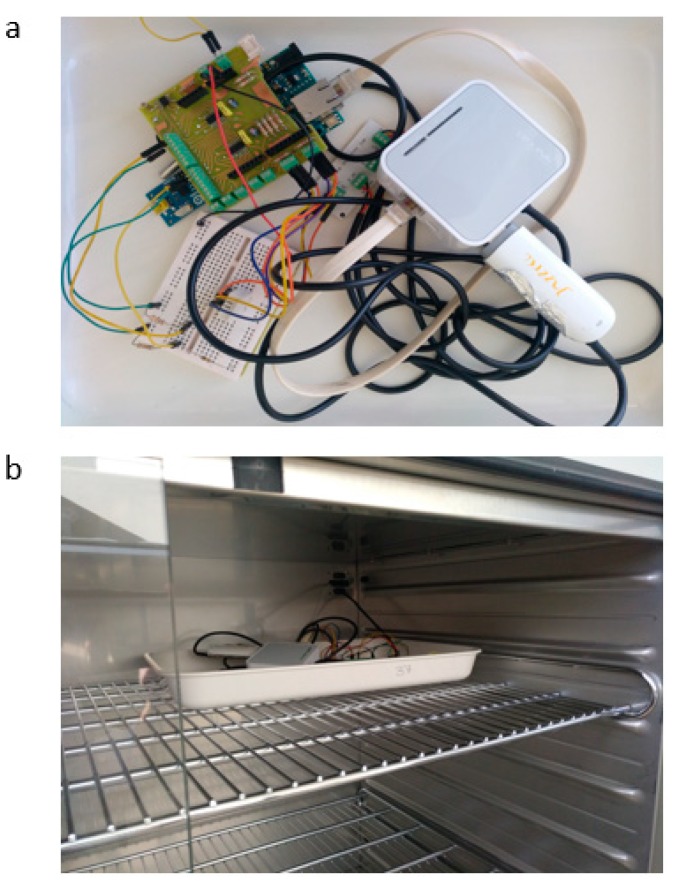
Prototype assembly used in all tests (**a**) and the prototype beingtested in the climatic chamber (**b**).

The temperatures of both the modem and router were measured. The temperature of the microprocessor was also monitored. DS18B20 digital temperature sensors from Maxim Integrated^TM^ (San Jose, CA, USA) were used for this purpose. Figure 7 shows the used configuration.

### 2.3. Results of Studying the System under Harsh Environmental Conditions

The exposure time of each cycle was 11 h (see Section 2.1.2 (c)) and the speed of temperature variation was 0.1 °C/min. The start temperature was the ambient temperature. The set-point temperature was 50 °C (Experiment #1, cycles from #1 to #10) and 55 °C (Experiment #2, cycles from #11 to #20). Table 4 shows the results of the measured temperatures.

**Figure 8:**
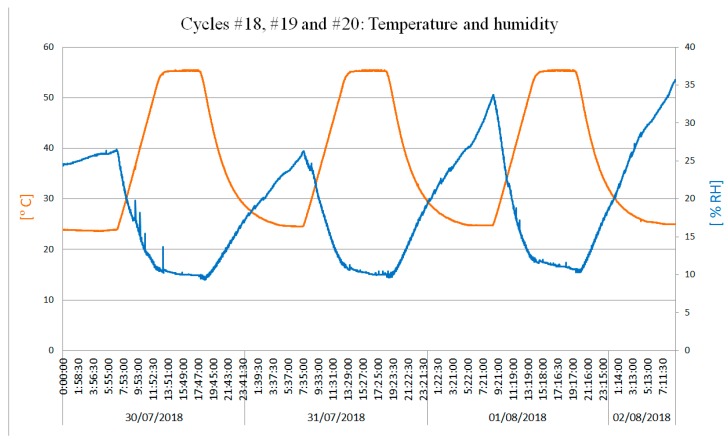
Temperature and humidity profiles: Cycles #18, #19, and #20.

Due to variations in the flux of air inside the climate chamber, the measured ambient temperature fell within the ranges of 47.2–51 °C (cycles from #1 to #10, set-point temperature of 50 °C) and 55.4–55.5 °C (cycles from #11 to #20, set-point temperature of 55 °C). The maximum router temperature measured was 63.81 °C and the maximum modem temperature measured was 68.25 °C. The datalogger reached 60.25 °C. Figure 8 shows an example of the temperature profile used in the test jointly with the %RH measurement. Gradient, temperature, and time values were defined using the experimental data (see Section 2.1.1).

The rise period was programmed according to the worst case of the recorded gradients, and the maximum temperature was reached in approximately 5 h. Then, the cycle was extended to complete 11 h. When the maximum temperature was reached, the chamber was disconnected, the cycle’s relaxing time started, and the system was driven to ambient temperature.

The main result of this experimental procedure was that the datalogger passed the temperature tests. During the 20 cycles, it correctly measured and did not stop sending data. Even with the most critical functions, such as connectivity and data transmission functions, which were the most temperature-sensitive functions (see Section 2), it operated correctly.

The results of the three dataloggers’ three-year uninterrupted operation under real conditions were excellent. Only one equipment failure occurred (the datalogger installed in Linares on 2 August 2018), which demonstrates the system’s reliability. On that day, the ambient temperature was 41 °C. The temperature inside the electrical cabinet (where the datalogger was installed) went above 48 °C.

## 3. Long-Term Performance in Harsh Environment

In order to check the system’s performance under real experimental conditions, monitoring systems in uninterrupted operation measured electrical and climatic conditions every 30 s. This test provided information on the system’s performance in the presence of different humidity levels, temperatures, or dust (among other variables).

### 3.1. Dataloggers under Real Climate Conditions

Apart from the temperature tests, different versions of the monitoring systems were installed to test their performance under real climate conditions. The systems sent data every 30 s. Table 5 describes the tested systems, their locations, and the time periods they operated in.

**Figure 9:**
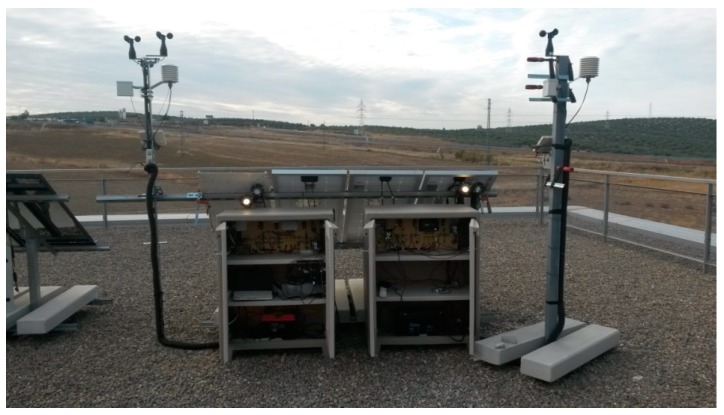
Two of the dataloggers installed in Linares: Datalogger SD version and Datalogger with a 3G connectivity version.

Dataloggers monitor a real SHS. In the three cases, the PV system included an 80 W mono-crystalline module, a 12 V lead-acid battery (90 Ah-C100), and a PWM serial charge controller. Figure 9 shows two of the dataloggers installed in Linares.

### 3.2. Long-Term Performance: Results

The installation of measurement systems allowed for the evaluation of the real behavior of the dataloggers and the recording of the possible errors that could appear after working under real conditions. As stated before, different types of failures were identified: Access failures, power failures, network failures, and equipment failures. After extending the experimental period to three-years under adverse weather conditions, a few external failures were recorded, such as power shortage or server drops that hindered the data storage).

The experimental procedure also allowed for the effect of expositing the system to adverse conditions in order to be studied. Deterioration was mainly detected in sensors. The temperature and humidity sensor installed in Madrid broke. The sensor correctly measured temperature, but the humidity measurement was wrong: The relative humidity was 100% even on sunny days.

**Figure 10:**
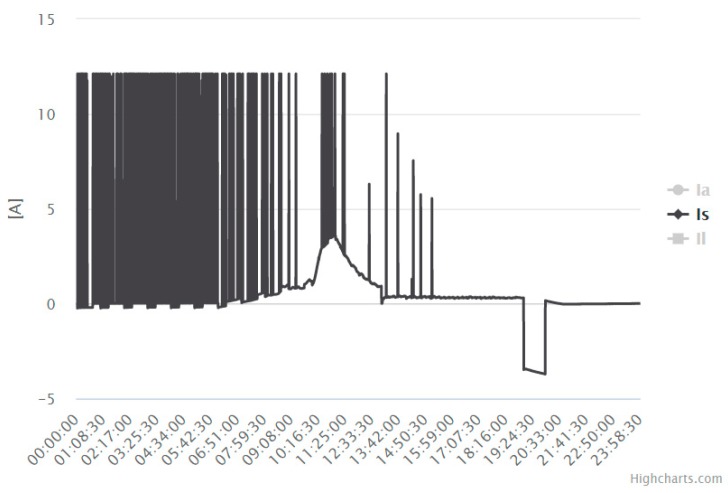
Battery current measured in Linares (24 June 2018).

For the datalogger with 3G connectivity installed in Linares, the current sensor did not measure correctly. The sensor’s measurement was correct, but some peaks often appeared, as shown in Figure 10. Both sensors (humidity and current sensors) were replaced. As the system was designed using low-cost sensors, replacing damaged sensors had no economic impact.

Very few failures were recorded in all the dataloggers and the origin of most of them was external (power shortage, server falls, etc.), or for the damaged sensors, failures were due to usual system deterioration when installed outdoors. The experimental test showed that the datalogger was the most reliable and robust; it has been demonstrated that the datalogger is designed especially to work correctly under harsh conditions.

## 4. Addressing Communication Failures—Adding Redundancy to the System (SD)

López-Vargas et al. [20] installed a low-cost datalogger in Oaxaca; these authors recorded network failures with a certain frequency and these failures led to a loss of data during the faulty period. As a future improvement to avoid the loss of data, they proposed implementing a backup system for data storage.

To help monitor PV systems in remote areas of developing countries that lack electricity and wired telecommunication networks, a datalogger being independent of an external source (e.g., laptop) is crucial [24]. Hence, the use of an SD as a backup unit for data storage proved to be the best solution. However, due to the limited memory [25] of the Arduino^TM^ UNO, integrating additional software to implement the storage process in an SD card as a backup system was not feasible.

An advanced board was selected as a solution. Arduino^TM^ MEGA was presented as the best option given its extended memory and its compatibility with hardware improvements [19]. All the hardware designs were adaptable to the new platform. Table 6 shows the comparison made between the Arduino^TM^ UNO and Arduino^TM^ MEGA boards. Every minute, in second 0 and second 30, parameters were measured. Every 30 s, after measuring and transmitting data, data were stored in the cloud and the SD card (backup).

### 4.1. Integrating a Backup Data Storage System

To avoid loss of data if failures occurred (network, access, etc.), a backup system based on a micro SD card was integrated into the datalogger to prevent the loss of data by storing the measured parameters if a network failure occurred during disconnection periods.

Due to the change in platform (that implies a change of processor, as shown in Table 6), the optimized datalogger was installed in Linares, Jaén, Spain (latitude N 38.085°, longitude W 3.646°), to study the behavior of the system under harsh environmental conditions. The datalogger measured ambient temperature, modem temperature, and router temperature (digital Dallas sensors). Every 30 s, these parameters were sent to a server located in Jaén and tothe cloud.

### 4.2. Results of the Datalogger with a Backup Storage System

**Figure 11:**
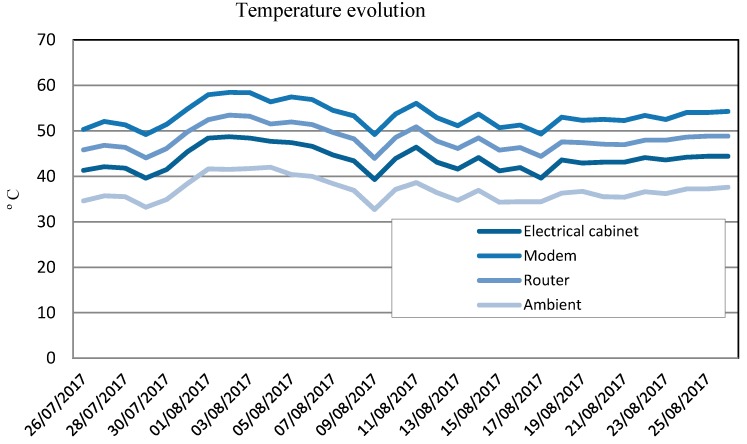
Temperature evolution measured by the datalogger designed around the platform Arduino^TM^ MEGA with a backup system of storage in Linares (Jaén, Spain) in August 2018.

The datalogger with the backup storage system based on an SD card was installed on the rooftop of the EPS Linares, Jaén, Spain (latitude N 38.085°, longitude W 3.646°). In the summer of 2018, this datalogger ran continuously. Figure 11 shows the temperature evolution recorded in the hottest month of the year in Jaén.

During this period, ambient temperatures reached above 40 °C. The datalogger was installed inside an electrical cabinet, where the measured temperatures were higher: The datalogger worked at operating temperatures over 48 °C. The datalogger measured correctly. No failures were recorded for either sensing or connectivity functions.

## 5. Discussion

During this work the evaluation of a datalogger for the measurement of autonomous photovoltaic systems installed in rural areas of developing countries [19] has been carried out. A connectivity version of the datalogger was installed in different locations [20] and various failures were detected: Access failure, network failure, power failure, and equipment failure. The equipment failure left the datalogger installed in Linares inoperative and it was attributed to the effect of high temperatures. Schelling et al. [17] detected that climates that are extremely hot, dry, and dusty can affect the productivity of the PV system and create additional wear and tear to the equipment. The highest temperatures (between latitudes N 35° and S 35°) include many of the developing countries and extreme climatic conditions can affect the correct functioning of dataloggers installed in rural areas of developing countries. Zubair in 2010 [26] stated that high temperature and excessive humidity are responsible for degraded performance and failures of many biomedical devices. Thus, in alignment with these studies, a thermal evaluation of the monitoring system was of special interest as well as the evaluation of the system installed in different sites (with different climates). After evaluating, the results demonstrated that the datalogger is highly reliable and robust, enduring harsh environmental conditions. Due to the quality of the wired networks [20], it is common for developing countries to suffer successive Internet drops. Thus, the monitoring system based on transmission via mobile communications is more reliable than a data acquisition system that is wired network dependent. Nwofe [27] in 2016 reviewed the challenges of mobile communication access in a developing economy, studying with a major emphasis on the Nigeria scenario. He detected a poor quality of service due to communication drops caused by shadowing and handset battery power failure, among other factors. In addition to the lack of quality of the networks, we also faced other problems such as possible access failures to the server [20] that would result in a loss of data, which could be solved with a redundant data storage system with a high recording capacity. A backup system was developed and tested in this work, allowing for the installation of the new prototype in rural areas of developing countries.

## 6. Summary and Conclusions

The low-cost datalogger intended for stand-alone PV systems in developing countries met the accuracy requirements established by the IEC61724 standard regarding the photovoltaic monitoring systems [15] and the integration of 3G connectivity with storage in a dedicate server and the cloud [16] was satisfactory. To study the reliability of the measurements, a high-accuracy commercial datalogger was used as a pattern [15].

Given the extreme climate conditions in many developing countries, the datalogger had undergone different performance tests. Three different versions of the datalogger were installed at different sites. These monitoring systems were installed to take continuous measurements and they worked from the date they were installed to the present-day, uninterrupted. Very few errors were recorded. The origin of all failures, except one, was external and not linked to the datalogger: Network, power, and access failures. One equipment error was recorded and attributable to high temperatures.

The equipment failure was registered in Linares. This error left the datalogger inoperative, and as it was attributed to the effect of high temperatures. This work focuses on the study of the reliability of the system by subjecting the datalogger to harsh temperature conditions.

The robustness of the system was proven by two different tests: First, the datalogger was subjected to extreme temperatures under controlled conditions while working under real conditions. For the first test, the datalogger passed the temperature tests designed (applying the worst conditions measured) in a confined space. During 20 cycles, the datalogger measured well and it did not stop sending the data. The most critical functions, the connectivity and data transmission functions, passed the test. It was demonstrated that the datalogger is highly reliable [28,29], measuring correctly even at 55 °C.

The second test (long-term performance) comprised three-years of continuous operation at the different sites. After uninterruptedly operating under real conditions for three-years, the datalogger recorded all the parameters in an uninterrupted way—demonstrating the reliability of the system under real and uncontrolled climatic conditions. The results demonstrated that the datalogger is robust and endures extreme conditions. The experimental procedure allowed for the study of the effect of exposing hardware to adverse conditions with deteriorating sensors: Wrong humidity measurements due to DHT sensor breaking and peaks in current measurements due to the hall-effect current sensor breaking. Low-cost sensors were selected for the integration in the monitoring systems. After operating for three-years, the replacement of two sensors (8.98€) can be considered as a minimum maintenance cost.

The meteorological and electrical measurements accomplished the accuracy requirements established by the IEC standard. To avoid loss of data, a redundant system based on an SD card was implemented, requiring the change of processor due to the limitation of memory. This improvement accounted for an increase in the final cost of only 16€. This latter datalogger version with a backup storage system based on an SD card was installed in the rooftop of the EPS Linares, Jaén, Spain (Latitude N 38.085 °, longitude W 3.646°). In the summer of 2018 this datalogger was continuously running. The datalogger worked at operating temperatures over 48 °C. As no failure has been detected to date, we can conclude that this datalogger version is robust and very reliable.

## Figures and Tables

**Table 1:** Climatic parameters measured in Linares between 1 June 2017 and 28 June 2017.

	Ambient Temperature [°C]			Humidity [%]			Electrical Cabinet Temperature [°C]		
Date	Minimum Value	Average Value	Maximum Value	Minimum Value	Average Value	Maximum Value	Minimum Value	Average Value	Maximum Value
1 June 2017	19.3	27.18	36	14.2	32.87	55.9	20.62	31.35	42.63
2 June 2017	20.5	27.88	36.2	21.1	36.58	57.6	21.5	32.31	43.69
3 June 2017	20.7	26.82	32.8	12.4	35.56	59.1	21.5	31.02	39.75
4 June 2017	19.1	24.76	29.5	13.9	32.57	55	20	28.74	36.88
5 June 2017	15	23.33	30.1	19.1	34.91	59.3	16.5	27.71	37.44
6 June 2017	16.7	24.28	32.1	19.7	32.93	55.6	17.19	28.75	39.06
7 June 2017	18.4	27.64	36.7	15	26.63	41.3	19.62	31.49	43.94
8 June 2017	19.6	28.97	37.4	14.1	23.67	38	21	32.71	45
9 June 2017	21.5	27.51	34.3	16.4	31.45	49	22.5	31.6	41.81
10 June 2017	19.2	29.28	38.2	16.7	28.76	49.3	20.94	33.51	45.5
11 June 2017	21	30.56	39.1	15.9	28.41	49.6	22.12	34.5	45.06
12 June 2017	21.7	31.02	38.8	13.3	21.17	34.9	22.37	34.38	44.5
13 June 2017	21.6	30.89	39	16.6	27.2	47.8	22.5	34.82	46.56
14 June 2017	20.9	29.99	40.2	16	41.05	70	22	34.67	47.56
15 June 2017	22.7	30.77	41.3	17.1	39.37	64.9	23.81	35.27	48.69
16 June 2017	27.2	33.29	42	16.2	26.52	38.4	28.19	36.65	48.69
17 June 2017	24.9	32.59	41.5	15.5	27.32	42.8	25.5	36.76	49.31
18 June 2017	24.5	32.19	40.3	15.3	25.75	42.1	25.5	36.38	48.19
19 June 2017	22	30.43	39.1	15.7	31.5	57.7	22.87	34.05	47.69
20 June 2017	23.4	31.35	39.5	15.7	28.49	44.6	24.06	35.42	46.81
21 June 2017	23.4	31.08	39.9	14.5	29.95	42.5	24	35.42	47.75
22 June 2017	22.1	30.08	38	20.1	41.88	74.3	23.06	34.67	45.88
23 June 2017	22	30.22	38.1	6.1	31.26	58.9	22.69	34.49	45.44
24 June 2017	21.4	31.61	40.7	14.3	21.46	36.3	22.44	35.25	47.63
25 June 2017	24.2	30.34	37.4	18.6	26.32	36.5	26.19	32.71	43
26 June 2017	20	27.67	34.2	12.9	35.2	66.1	21.44	31.66	40.44
27 June 2017	18.6	24.69	30.8	17.3	34.17	61.7	19.69	28.44	36.63
28 June 2017	18.1	23.67	28.2	27.7	43.84	64.7	19.19	27.54	34

**Table 2:** Extreme ambient temperature (>40 °C) measured in Jaén in 2017.

Day	Month	Average Temperature [°C]	Maximum Temperature [°C]	Minimum Temperature [°C]
16	June	33.2	40.8	27.3
17	June	33.8	41.2	28.1
24	June	32.4	40.2	23.8
11	July	31.6	40.2	23.2
12	July	34.4	43.5	25.3
13	July	36.9	44.7	27.2
14	July	35.9	44.1	26.3
18	July	33.2	40.2	26.2
28	July	32.8	40	24.2
29	July	32.7	40.1	22.8
30	July	32.9	40.2	24.1
4	August	33.1	42.1	23.4
5	August	35	41.2	27.1
6	August	34.1	40.4	24.9
7	August	33.3	40.1	26.2
19	August	32.9	40.2	24.2
20	August	32.8	40.7	25.2
20	August	32.8	40.7	25.2

**Table 3:** Final values selected for the tests.

Parameter	Experiment #1	Experiment #2
Maximum temperature	50 °C	55 °C
Speed of variation of the temperature	0.1 °C/min	0.1 °C/min
Exposure	11 h	11 h
Number of cycles	10	10

**Table 4:** Temperature measured in the tests.

Cycle	Date	Maximum Modem Temperature [°C]	Maximum Processor Temperature [°C]	Maximum Router Temperature [°C]	Maximum Ambient Temperature [°C]
1	29 May 2018	54.94	52.06	55.25	47.2
2	30 May 2018	56.56	55.38	55.75	47.2
3	31 May 2018	59.19	57.44	59.13	50.2
4	1 June 2018	59.13	57.5	59.13	50.2
5	4 June 2018	59.19	57.5	59.13	50.2
6	29 June 2018	59	54.81	58.69	50.2
7	2 July 2018	59.69	54.81	58.56	50.2
8	3 July 2018	59.63	54.81	58.5	50.5
9	4 July 2018	60.56	54.88	59	51
10	17 July 2018	62.13	55.56	59.13	50.8
11	18 July 2018	67.12	60.19	63.75	55.5
12	19 July 2018	67.56	60.19	63.81	55.4
13	23 July 2018	66.81	60.19	63.63	55.4
14	24 July 2018	67.25	60.25	63.69	55.5
15	25 July 2018	67.19	60.19	63.63	55.5
16	26 July 2018	67.31	60.25	63.69	55.5
17	27 July 2018	66.56	60.25	63.63	55.5
18	30 July 2018	67.19	60.19	63.63	55.5
19	31 July 2018	67.5	60.25	63.69	55.5
20	1 August 2018	68.25	60.25	63.69	55.5

**Table 5:** Dataloggers installed and tested under real climatic conditions: Versions and locations.

Version	Location	Time of Operation
Datalogger SD [19]	Linares, Jaén, Spain. Latitude N 38.085°, longitude W 3.646°	3 years
Datalogger with 3G connectivity [20]	Alcalá de Henares, Madrid, Spain. Latitude N 40.513°, longitude E 3.339°	3 years
Datalogger with 3G connectivity [20]	Linares, Jaén, Spain. Latitude N 38.085°, longitude W 3.646°	3 years
Datalogger with 3G connectivity and SD backup system	Linares, Jaén, Spain. Latitude N 38.085°, longitude W 3.646°	8 months

**Table 6:** Arduino^TM^ UNO and Arduino^TM^ Mega 250 comparison.

Name	UNO	MEGA250
Processor	ATmega328P	ATmega2560
Operating/Input Voltage	5 V/7–12 V	5 V/7–12 V
CPU Speed	16 MHz	16 MHz
Analog In/Out	6/0	16/0
Digital IO/PWM	14/6	54/15
EEPROM [kB]	1	4
SRAM [kB]	2	8
Flash [kB]	32	256
USB	Regular	Regular
UART	1	4
Price [€]	19	35
